# Odontological Society of Pennsylvania

**Published:** 1894-06

**Authors:** 


### ODONTOLOGICAL SOCIETY OF PENNSYLVANIA.

At the regular monthly meeting of the Society, held February 10, 1894, President Dr. Darby in the chair, upon the conclusion of routine business, the President called on Dr. George S. Allan, of New York, the essayist of the evening.

Before reading the paper, the doctor exhibited two dental appliances, and, in exhibiting them, said,—

"What I wished to show you will be a partial failure. I had asked to have four storage batteries here, but find four gravity cells instead. It is a very compact and valuable electric mouth-lamp, and thinking it might interest you, I brought it over. The voltage supplied by these cells is not sufficient, but may give you an idea of its value. By means of this rheostat,—which is a very small one, and can be made for six or six and a half dollars,—you can regulate the current perfectly. The lamp itself would cost about three dollars and a half. When the current is on, you can, by the aid of this little lever, get a flash light in the mouth. If the lamp is broken it is easily replaced at a minimum cost.

"The other matter that I brought over is one that I showed to Dr. Kirk a year or so ago, and tried to get him to take an interest in it, but he did not do so at the time, and so I brought it over to see what the members of the Pennsylvania Society thought of it. It is a little pan for annealing gold. Oftentimes you have a surplusage of cohesive gold after finishing an operation, and it is awkward to take it up piece by piece. The pan is composed of fine wire gauze. It cuts off the gases on the principle of the Sir Humphry Davy lamp. You can regulate the heat in annealing gold without injuring it, and the gold is annealed in mass instead of piece by piece. I find it answers the purpose."

Dr. Kirk, in response, said, "If Dr. Allan had been at the Columbian Dental Congress, he would have seen a lamp which I think will supersede any of the mouth illuminators we have yet been shown, one which is certainly a unique contrivance. It consisted of a small illuminator, such as has been shown you, but in front or back of it was a parabolic reflector, made of vulcanite and brightly polished on the inside. The lamp was set in about that way [illustrating it as sheathed in the reflector]. The encasement was carried around in front, and in a socket towards the front was set a glass rod which could be bent in any shape, curiously enough, almost at right angles. This end [illustrating] perfectly plain, and so fixed that the light was reflected or condensed from this parabolic reflector and thrown into the end of the rod and conducted down the rod. The great advantage of this electrode was that it could be bent in any direction, and it could be carried to any spot in the mouth, the advantage being that the rod never became heated, and the illuminating power of the lamp was something remarkable. It made the diagnosis of pulpless teeth perfectly feasible, and has been used for determining the question of the collection of fluids in the antrum. I think that is an improvement in the idea Dr. Allan has shown here. I have sent to Paris and tried to get one of the lamps. The difficulty so far has been the fact that the amount of heat collected and thrown against the bulb of the lamp is sometimes great enough to soften the lamp. For the purposes of illumination I think it is the best arrangement I have ever seen. The arrangement for annealing gold is not satisfactory. He says that the wire gauze cuts off the gases. It cuts off the flame the gases go through."

*Dr. Truman.*—How about the safety lamp?

*Dr. Kirk.*—The gas goes through, but it does not ignite.

*Dr. Allan.*—I won't discuss the question, except to say it works satisfactorily. It anneals gold, does not overheat it, and the annealing is as perfect and complete as with a spirit-lamp.

As to the lamp, what Dr. Kirk refers to it does not affect the construction of this one. It is only something added to it. Further, this one you can obtain, and the other you cannot. This gives ample light and aids greatly in making a correct diagnosis in many doubtful cases. The lamp I intended to bring over was, unfortunately broken just as I was coming away. It has a little vulcanite back to it so that the side that is to be next to the tongue is protected, not only by the rubber shield, but the shield is set in plaster and asbestos, so that you can use the lamp quite a little while before the heat penetrates the plaster and asbestos. It only takes half a minute or a minute to make an examination of a pulpless tooth, so that the heat will not be perceptible.

(Dr. Allan next exhibited some cases illustrating appliances for holding the teeth in place.)

The first represents the case of a patient where a tooth had been brought in place by an elastic, and after a few days a gold plate was made and fitted to the palatal surface of the teeth and held in place by three little pivots attached to the plate and cemented in position. The tooth is now held firmly in position. The patient was about fifty-five years old.

The next represents a case where a second molar was loose. The patient has worn the brace something like four years, giving much comfort and prolonging the life of the tooth. Without it, in a day or two the tooth becomes sore, and if it were removed the tooth would soon be lost.

In the use of sulphuric acid in a ten per cent. solution, with this little platinum spatula, I find I can remove tartar very easily. Ever since Dr. Kirk put in my hands some asbestos fibre, which is as fine as cotton, I wrap a little of it around the blade, and I can carry the asbestos fibre, after dipping it in the acid, around the roots in places that I could not reach in any other way, and by a little mechanical rubbing or abrasion with the asbestos fibre and sulphuric acid the tartar is loosened very rapidly. With this and ordinary chisels you can go over a mouth and remove at least nine-tenths or all of the tartar with it and not injure the gums in the slightest degree, and certainly not the alveolar process.

Dr. Allan then read his paper on "Pyorrhœa Alveolaris."

(For Dr. Allan's paper, see page 219.)

DISCUSSION.

*Dr. Peirce.*—I have prepared a paper to read this evening, but as it is late, and I desire to hear from others, I will occupy but a few moments. I would like to ask Dr. Allan if he presents these casts as representing cases cured through local treatment? [Dr. Allan answered in the affirmative]. I never in the paper to which the essayist has devoted his criticisms made a single allusion to what is called, and recognized from its color, black tartar. I never made a single allusion to the tartar that was deposited from the saliva—which the black evidently from its location is—as being the cause of pyorrhœa alveolaris. I did not say that black tartar was not deposited from saliva, but believe that it is, as every one who recognizes its location must. I never saw a case of pyorrhœa occasioned by black tartar; inflammation of the gingival borders of the gums is the extent of its disturbance. It is entirely different from the deposit examined by Professor Congdon, of the Drexel Institute. Different in its location, structure, and color; different in its origin.

Certainly there is no one here who has not recognized the presence of black tartar and its local influence on the gum. It never goes beyond the fifth of the length of the root towards the apical end; it is confined to a locality just under the gum margins.

The tartar examined by Professor Congdon was taken directly from or near the apical end of the root, and where there could be no connection with the gingival borders whatever. His specimens were deposited by or from the plasma of the blood, and could not have come from the saliva, because the locality and structure prohibited intercourse between the parts. The product from the plasma of the blood was what Professor Congdon analyzed, and it was his critical analysis that was published in the paper to which allusion has been made. Dr. Allan says he is not familiar with any one who has taken similar views with myself. I would like to read an extract from a paper placed in my hands two days since by Dr. Faught, which says,—

"I am of the opinion, however, that the deposition of the concretions upon the roots of the teeth in those localities not easily reached by the saliva, or in which the presence of the saliva would be an impossibility, is due to the causes which produce the chalky formations found in the joints and fibrous tissues of gouty and rheumatic individuals.

"The thought has occurred to me, though I have not had time to demonstrate it positively, that the concretions found upon the roots of the teeth in the locations just named were masses of urate of soda with phosphate and carbonate of calcium, and that they are deposited directly from the secretions, as is often the case in rheumatic arthritis."

Furthermore, it would seem that these concretions were the cause of the inflammatory condition rather than the result of it; in proof of this let me state that clinical observation teaches that suppuration often occurs about the roots of the teeth at remote points from the gum margin, and which have no outlet until the pericementum is dissected from the roots of the teeth by the accumulation of pus. I have seen cases in the lower jaw in which an abscess had been formed upon the roots of living teeth, between the neck and the apex, and in which the attachment of the gum at the neck of the tooth was intact, and the pus did not escape until nature had perforated the soft tissue, or relief was given by the use of the knife. In such cases I have never failed to find concretions upon the root at the point of suppuration; and this could not possibly have been deposited from the saliva, and it is fair to presume that the deposit upon the root was the source of irritation that produced the abscess, rather than that the inflammatory condition was produced by some remote cause, and the formation of the deposit the result of the inflammation.

Phagedenic pericementitis is sometimes directly traceable to a rheumatic condition of the system or the uric-acid diathesis. In several cases in which I have analyzed the urine, uric acid was found largely in excess of the normal quantity.

In all of the cases which I class as rheumatic, concretions are found upon the roots of the teeth, and many times in locations which preclude the possibility of salivary origin.

Under restricted diet, in which meats, wine, and malt liquors are cut off, there is soon a marked diminution in the quantity of uric acid excreted, and an equally marked improvement takes place in the symptoms manifested in the oral cavity, which cannot be accounted for by the removal of the concretions and local treatment alone. In one case, which has been under observation four years, the periodical aggravation of the oral symptoms are a sure indication of the presence of an excessive amount of uric acid in the urine, and as soon as this condition is corrected the inflammatory conditions of the pericementum are greatly relieved. Local treatment is necessary, but this alone is not sufficient; we must strike deeper and correct the morbid condition of the system if we hope to effect a cure.

That is the testimony of Dr. John S. Marshall, of Chicago, who says he has seen cases where there has been deposit without destruction of the gum margin. It was only last week or within a few days that a patient came to me from Dr. Mary H. Stillwell, of this city, where the left cuspid tooth was as vital as a tooth could be, having an abscess opposite the apical end, and on this end there was a rough incrustation. The pulp was undoubtedly alive.

*Dr. Allan.*—No sinus?

*Dr. Peirce.*—No trace of a sinus or pocket. I have seen three cases exactly like it,—teeth where there has been an abscess and the tooth retained its vitality.

*Dr. Allan.*—You didn't know anything about the end; you didn't extract it?

*Dr. Peirce.*—No, but I examined it without an instrument.

*Dr. Allan.*—Was the incrustation there before the abscess formed, or did it come afterwards?

*Dr. Peirce.*—I assume it was there before the abscess was established, and was to an extent the cause of it.

*Dr. Truman.*—What evidence have you that the pulp was alive in that tooth?

*Dr. Peirce.*—It was not off-colored when there was a strong light thrown upon it; one cannot easily be deceived. It is as certain as it is possible to ascertain anything. Besides, the abscess healed without disturbing the pulp-chamber or canal. Now, in regard to another point. I have in the last three months had twelve cases of pyorrhœa under my charge, and with the exception of three occurring in school-teachers, every one had other indications of gout. There are gentlemen here to-night who can give stronger evidence than this. The association of pyorrhœa with gouty diathesis is certainly so true that it cannot be gainsaid. Let me say that Dr. Allan's paper may be all true from his stand-point. If he did not take the product from blood plasma, he could hardly expect to find uric-acid compounds. Tartar on the apical end of the root is not black tartar, but is of a very different composition. Tartar found in that locality usually comes from the blood, while that on the crown and neck is undoubtedly from the saliva.

*Dr. Allan.*—Referring to this same article of Dr. Peirce's, I find on page 2 the following. But first I would say that in that article he failed to draw attention to a third kind of tartar. In my paper I did not deny that there were cases at times which seemed to point to some other causes of pyorrhœa alveolaris than the purely local one at the neck of the tooth. In his paper, Dr. Peirce refers to a special kind of tartar. In his article in the INTERNATIONAL DENTAL JOURNAL, he says, "In the first place, I believe that while pericementitis is associated with calcic deposit, the origin of the calcic salt and the antecedent condition which determines the locality and character of the deposit as well as the train of totally distinct symptoms which follow, lead inevitably to the conclusion that two different diseases have thus far been confounded. In one form of pericementitis the origin of the calcic salt is the saliva, and in the other form, the blood." The calcic deposit Dr. Peirce refers to and the tartar of Dr. Black are one and the same thing. So I was perfectly right in presuming he drew attention to the same kind of tartar referred to by Dr. Black. He says, "The former I shall designate as ptyalogenic calcic pericementitis, expressive of the idea that in its origin it is local, peripheral, and salivary. The latter I shall designate as hæmatogenic calcic pericementitis, expressive of the idea that in its origin it is constitutional, central, and associated with some modification of the normal composite of the blood plasma." That is the serumic tartar of Dr.

Black. Dr. Peirce fails to draw attention to a third kind of tartar. Now, here he says, "I have had some specimens of this tooth deposit examined by Professor Congdon." A person is very easily led astray if the doctor cannot be more definite, and I can only say that what he refers to is the ordinary black deposit. It is a cause of pyorrhœa, but not the only cause by any means. I have seen cases of pyorrhœa where there was no deposit of any kind that I could detect.

*Dr. Kirk.*—My knowledge of this particular phase of the subject arose two days before I had a conversation with Dr. Peirce upon it. My attention was called to a case that had been sent me over six months ago by Dr. Harrison Allen, for relief of pyorrhœa alveolaris, to see if I could do something to relieve the distress. It has now been over six months since I had the case under observation, extending over three or four treatments. When the patient first came to me the lower incisor teeth and two of his bicuspids, as nearly as I could recollect, were affected with pyorrhœa in a form almost as bad as the worst case I ever saw. I told him frankly that I believed it was only a question of a short time before he would lose his teeth; that what I should do would be simply palliative, and would only put off the evil day. As carefully and thoroughly as I could I removed the deposits from the roots of the teeth, and after applying the ordinary antiseptics and stimulating treatment which we give these cases, I dismissed him.

I saw him six months or so afterwards, which was two days before reading Dr. Peirce's paper before the Odontological Society of New York. I noticed that as he came up my stairs he looked brisk, well, and happy, whereas, before, he had the appearance of a chronic invalid. I examined the mouth, and it was as healthy and as clean as any mouth I ever saw. There was a restoration of the tissue, and the mouth was in splendid order. I have treated a great many cases, but never saw such a result. I knew as soon as I saw him that my treatment had not done it. I asked him what he had done. He told me that he had been under treatment all his life for various ailments with but little benefit, but Dr. Allen had put him under a strict gouty *régime.* When Dr. Peirce was relating the substance of his paper to me I reported this case to him as one which, in my belief, illustrated perfectly the truth of his theory.

It seems to me that the principal point at issue to-night hinges upon the analysis of these concretions and their origin. Two methods of analysis are applied to the investigation of tartar. One by which the approximate constituents are determined, and those by which the ultimate constituents are determined. The analyses quoted by Dr. Allan are of the sort in which the ultimate constituents alone were shown. If you pay attention to the report of results of the analyses you will observe that the tartar contained so much organic matter, so much water, and so much carbonate of calcium. If any uric acid or urate of calcium had been present, it would in this case be measured in terms of organic matter and calcium carbonate. In other words, it would not have shown uric acid under the circumstances, even if any had existed.

The analysis of tartar which Dr. Allan sent to Professor Rickets is also faulty in this particular, that it had been collected from pyorrhœa cases with open pockets, and it is tartar of an indeterminate sort; probably a mixture; and yet that analysis did show uric acid present. I have not had a case so far in which I could determine the question whether there is a deposit of concretions around the roots of teeth without some breaking in the gum margin. I believe there is. Personally, I have not made the observation. Dr. Allan says he never has seen a case where there is concretion around the roots of the teeth, without a break in the gum margin; that all cases which he has examined have formed from the gum margin downward. Of course that may be true, because those are the only cases we have been taught to observe.

I have a tooth in my own mouth in which the pulp is still alive, and there is quite a bunch about that root resulting from alveolar abscess. I have four or five teeth which, if I am indiscreet in the matter of eating, become very sore; but since I have been taking especial care in a dietetic way and living within the limits of a gouty *régime* I have had no trouble.

I have some fifteen patients—pyorrhœa cases—at present under a strict gouty *régime*, and I hope to be able to report on these later.

I had, a few days since, an opportunity of observing in the case of a lady patient a gouty outbreak simultaneously with an acute attack of pyorrhœa inflammation, where the gingival margin around the teeth was much swollen and painful; her hands showed that she had the typical gouty condition; that she had just been passing through an exacerbation of gout with the acute pyorrhœa inflammation simultaneous with it.

*Dr. Thomas.*—Any one who has been the victim of impaired or perverted digestion will readily recognize the constitutional causes of gout and rheumatism. A few years ago I had occasion to go upon a milk diet for six months, not even eating a cracker in that time. Previously, while getting into the condition which made the milk diet necessary, I suffered greatly with rheumatic pains and gouty symptoms. After resuming the normal diet it was easy to discover what it was I had eaten that either increased or decreased the rheumatic or gouty symptoms.

There was no organic disease of the digestive organs, but their ability was impaired by reduced nerve-force, brought on by overwork. To eat at one time too much was as bad as to eat or drink articles of food which should be prohibited.

Dentists especially, but people in general, do not get out-door exercise enough to utilize the stronger foods, and should confine their diet to such as will be assimilated readily. This winter I have left beef entirely alone, and my general health was never better, and I have not had a twinge of rheumatism or gout. I have noticed that during the times when I had evidences of imperfect digestion that pyorrhœa always accompanied the condition, the same as gout and rheumatism. Since Dr. Peirce's paper has appeared I have taken pains to inquire of eighteen or twenty patients suffering from pyorrhœa if they ever suffered from rheumatism or gout, and they have all of them answered "yes;" and, upon further conversation, they have all claimed that beef was their principal animal diet, and they did not see how they could get along without it.

*Dr. Jack.*—Dr. Peirce's conclusions tend to establish on definite grounds two different pathological conditions of the peridonteum which heretofore have not been clearly differentiated.

In reference to phagedenic pericementitis, it has not escaped observation that thick proliferation of the peridental membrane not infrequently occurred attended by absorption of the alveolar process, but it has escaped my attention that deposits have occurred at the apex of the roots, unless some communication has been established either by fistulous tracts transversely through the tissues or along the course of the peridental membrane, and these cases have mostly occurred with dead teeth. I have, however, observed that in nearly every instance where proliferation of the peridental membrane has taken place it was associated with evidences of the existence of the gouty diathesis.

It is not infrequent to observe attacks of gouty inflammation of the peridonteum. Sometimes this structure, from its free supply of nerve-filaments, becomes highly sensitive when neuralgic pain occurs similar to that manifested in the earliest stage of simple pericementitis.

One case in particular is worth being mentioned in this connection of over fifteen years' observation. At times, more particularly after partaking of wine, the right central would become loose, which, on the second occurrence, was diagnosed to be of gouty origin, which then was questioned by the patient.

Lately a most severe attack occurred, when tumefaction had taken place, with considerable swelling of the gum, simulating the second stage of alveolar abscess. As the tooth was not responsive to alterations of temperature, and was a little off color, I decided to drill to the pulp-chamber to give relief to a condition which I had long feared would supervene. At the half-way distance sensibility was excited, proving a living pulp. This will be perceived to be as clear a case of gout manifestation as if the tumefaction of a joint had taken place. The patient now has large gouty concretions in the joints of some of the fingers.

In one other instance, where the patient was also of a gouty diathesis, distinct abscesses occurred on two occasions of the tumefied gum, and after the evacuation of a single drop of pus, which appeared to be formed in the superfices of the gum. This case was like the previous one with this exception, and also there was a shallow deposit of thin, dark salivary calculus beneath the margin of the gum.

*Dr. Truman.*—The subject has been pretty well handled, and a great deal written about many of its points, until it is somewhat tiresome. I want to express gratification that we seem to be approaching a period in dentistry when individuals have come to the conclusion to work upon matter in the laboratory rather than in their own brains, and it is hoped we will have a better class of papers and intelligence in regard to many things. I want to congratulate Dr. Peirce on his work, although I cannot agree with him in his conclusions. Possibly he may be right, and I and some of the rest of us wrong. He has demonstrated, however, and with a considerable degree of certainty, that uric acid may be found around the roots of the teeth. I cannot believe he has proved that pyorrhœa is a result of this. It may be at times. Dr. Allan has alluded to one point,—that pyorrhœa begins frequently at the apical extremity. We have had it repeatedly stated here to-night that that is true. Now, I think I have had a great deal of experience both private and clinical, and I must say that I never have seen a case that I could positively determine began at the apex of the root. I do not consider it possible from a pathological stand-point that this concretion should occur at the apex of any tooth, covering the point where the pulp enters the tooth, and that pulp remain alive. It seems to me an impossibility. The very fact that the concretions occur at the apex, and press upon the pulp, would destroy the pulp of that particular root. I know very well that with multirooted teeth we may have two of the roots alive, but the root that is particularly affected will be dead. I will just ask Dr. Peirce to examine one I have with me, and decide whether he would not think that such a tooth was a living tooth if examined in the mouth?

*Dr. Peirce.*—I should think the crown and buccal sides indicate vitality.

*Dr. Truman.*—Allusion was made in the paper to lymph or serum passing from the cementum into the dentine, and in reverse order back again to the pericementum. Now, I believe there is no question at all that fluid can pass into the dentine through the cementum, but as it passes out in reverse order, how can it affect only a certain portion of the pericementum. It should be deposited in the entire tissue. The urates will not disturb any given part unless there be a lack of nutrition in that particular point. In other words, according to the best pathologists, the tissue must have experienced a loss of tone before it can be affected by this gouty deposition. But we find pyorrhœa beginning at the gingivæ. If it begins there, it is in advance of the deposition, and therefore has had a beginning before the urates have been deposited.

If I understand it, we cannot possibly recognize the theory that uric acid is at the foundation of this disease. That it may have something to do with it may be true, but when I find that Dr. Peirce limits the beginning to thirty-five years of age, he is, in my opinion, giving the whole thing away.

I have been studying this subject for the last thirty years, and I am satisfied that pyorrhœa alveolaris does not depend solely upon a gouty condition. It is, in my judgment, independent of any of the deposits, and I cannot agree with Dr. Allan that these are usually found in pockets where pyorrhœa exists. The age limit will not, I think, hold. I have had a number of cases where the age did not exceed twenty.

I feel assured that this disease is mainly local. Constitutional conditions may be present sometimes to increase it. That it is local is evident from the fact that in all cases we can produce it. Take an individual with perfectly healthy gums and irritate them with clamps, ligatures, separators, etc., and nothing done to render the gum aseptic, there will be continued irritation and development of micro-organisms so extended that in forty-eight hours there will be incipient pyorrhœa. If this be true, and I cannot see how it can be controverted, it cannot be said pyorrhœa is always occasioned by constitutional difficulties.

Dr. Peirce, somewhere in his remarks, said that it is a disease that is rarely cured. If that were so it would be the opprobrium of dentistry. We had better give up our practice entirely. What! cannot cure a simple irritation of the pericemental membrane! I cannot believe it. I know it can be cured.

There came to me, a year or so ago, a very difficult case. The patient had been under the care of a prominent dentist who acknowledged that he could do nothing with it. All the lower teeth were so loose that their salvation seemed problematical. I, however, began the treatment as I understood it. This proved so effectual that in six months time he came and exhibited them to me, and with one exception they were as firm as any in the mouth. The gums were healed and perfectly adherent to the cervical border.

This brings another point. The cement in these teeth was practically dead. There was no pericementum upon them. It seemed impossible that bone could form around the teeth, but it did, exactly as I suppose it forms around implanted teeth. That pyorrhœa can be cured I entertain no doubt, but whether all cases can be is still an open question.

*Dr. Brubaker.*—Though not feeling myself qualified to speak either on the causation or pathology of pyorrhœa, there are several statements in the papers of Dr. Peirce which find confirmation in the field of clinical medicine, and to which I wish to allude.

As to whether a pericementitis which is followed by the phenomena which characterize a case of true pyorrhœa alveolaris begins primarily at the gum margins from a deposit of lime salts from the saliva, or whether it begins near the apical extremity of the root from a deposit of organic salts from the blood, is merely a matter of observation, and should therefore be solved by an attentive study of individual cases.

A matter of the utmost importance at the very outset of this discussion is a definition of terms, so that we may all know just what is meant by the term pyorrhœa. Much confusion and waste of argument may thus be avoided.

Dr. Peirce, in his several papers on this subject, takes the position, if I correctly understand his argument, that (1) a pericementitis attended by a flow of pus may be caused by a salivary deposit of calcium phosphates and carbonates, and (2) that a pericementitis attended by a flow of pus may be caused by a special hæmic deposit of calcium and sodium urates and free uric acid, but that the train of symptoms and pathological changes which follow these two absolutely different causes are so unlike in many respects that two distinct diseases are to be diagnosticated. If the sketch of the characteristic features of these two forms of pyorrhœa be correctly drawn, and if his observation can be substantiated by the observations of others, the justness of his differential diagnosis becomes self-evident. There is, I am told, a general consensus of opinion among dental practitioners that some cases of pyorrhœa are of short duration, yielding readily to treatment, and that other cases are of long duration, extending through months and years, attended by the absorption of the alveolar process and the eventual shedding of the teeth, and which are practically non-curable. Now, it is this latter form of pericementitis that I, in common with Dr. Peirce and other observers believe to be a local expression of the gouty diathesis, and as such to be treated along constitutional and specific lines; and my opinion is based upon the resemblance of this disease to other local expressions of the same diathesis occurring in other portions of the body. As to the chemical composition of the salts removed from the roots of the teeth by this chronic destructive form of pericementitis there is no doubt in my mind. I know from my own observation of six cases that they were uric-acid salts, and that they were not derived from the saliva.

The statement by Dr. Peirce, that every case of pericementitis which progresses to the stage of pus formation and discharge is not to be regarded as a case of pyorrhœa in the sense in which this term is generally understood, is, I imagine, accepted, by all who have had much experience with the disease, and who possess ordinary powers of diagnosis.

But that such a non-specific pericementitis may, by the addition of a uric-acid deposit, acquire those specific features which characterize true pyorrhœa is most probable. Analogous conditions occurring in all portions of the body may be readily cited confirmatory of this view. A non-specific catarrhal inflammation of the bronchial mucous membrane produced by atmospheric changes or disturbances of the pulmonary circulation, though lasting for a variable length of time, will usually yield to treatment directed to the nutrition of the membrane or to the circulation, as the case may be. Yet there is a certain number of cases which will resist all such medication. This fact, coupled with the observation that bronchitis was frequently associated with gout, induced Dr. Greenhow to investigate the possibility of a causal connection of these two diseases. From a study of a large number of cases he found that thirty-seven per cent. had various manifestations of gout. In summing up his investigations he states it as his conviction that there is an intimate relationship between the gouty constitution and chronic bronchitis. In many instances where gout has not developed into its characteristic forms it manifests itself in the form of chronic bronchitis. He further states that in many instances no doubt the disease (bronchitis) is first developed by some external exciting cause, though frequently by a much slighter one than would be likely to produce the same effects in a healthy subject. Crystals of uric acid have in several instances been detected in the sputum of chronic bronchitis of gouty people. A gouty pericementitis finds its counterpart in gouty bronchitis. Again, a simple non-specific pharyngitis may take on specific characters when occurring in gouty subjects. In all its symptoms a gouty throat is like no other. The mucous membrane of the palate and pharynx is red, glazed, œdematous, penetrated with turgid veins, and covered with a grayish secretion. In a case reported by Dr. G. de Mussy, masses of urate of lime and carbonate of lime were discharged from the mucous follicles of the pharynx. Inflammation of the parotid glands, of the general mucous membranes of the skin, of the nerves of the viscera may, when occurring in gouty people, develop specific features which would make their relationship incontestable. A gouty pericementitis finds its exact counterpart in a large number of local inflammatory conditions in various regions of the body.

It is therefore highly probable that when pyorrhœa occurs in gouty subjects its continuance is directly connected with uric-acid irritation. An objection to this view as to the pathology of pyorrhœa has been made on the ground that, as in some instances the patient does not exhibit any of the usual symptoms of the gouty diathesis, therefore it cannot be associated with it. Now, the amount of consideration which this objection should receive will depend on the amount of attention the author of the objection has given to a study of the pathology of gout and upon his ability to diagnose the multiform phases of this extraordinary constitutional condition. When it is remembered that the gouty manifestations are often so subtle and obscure that even the most expert are baffled, is it to be expected that all dental practitioners not familiar with general pathological conditions should be capable of detecting their presence and influence? Yet I have no doubt that even a most superficial examination of the various organs and tissues of the body will reveal some gouty condition in all cases of true pyorrhœa.

As in the practice of general medicine, so I presume in the practice of dental medicine, the specific cause of a disease is frequently determined by the results which follow specific treatment. If some local persistent inflammation of bone, skin, or viscus, or some chronic form of neuralgia, or some peculiar eruption, which resist all local and constitutional treatment, should yield and disappear under some specific remedy, as mercury, quinine, colchicum, and potassium, there would not be the shadow of a doubt that there had been present some remote manifestation of the syphilitic, malarial, or gouty diathesis. Indeed, this is regarded as proof positive in the absence of all other evidence that the local trouble was but an expression of a constitutional disorder.

Now, it occurs to me, from a consideration of the histories and the results of the specific treatment instituted by Dr. Peirce, that we have all necessary proof that pyorrhœa alveolaris is a specific pericementitis caused and maintained by the deposition of uric acid and its salts from the blood into a pericemental membrane, the nutritional level of which has been lowered by some local mechanical or chemical cause.

*Dr. Burchard.*—Any remarks I can make would but serve to corroborate the statements and opinions of Dr. Brubaker. Dr. Allan has said, or quoted as authority Dr. Draper's article in "Pepper's System of Medicine," that the gouty diathesis is not necessarily caused by the presence of an increased amount of uric acid or urates in the blood. This is, on the other hand, regarded by Garrod, Niemeyer, Duckworth, Ebstein, and others as the distinguishing feature of this particular disorder. There is no other means of explaining the phenomena of the disease; even the trophoneurosis theory is the same element involved. Further, one would infer from Dr. Allan's statement that we are to accept the presence of uric acid or urates as an expression of an incomplete retrograde tissue metamorphosis; whatever view we may take as to this, the weight of evidence is assuredly in favor of the production of these substances being through a faulty metabolism. Lauder Brunton's writings upon the function of the liver strengthen such an assumption.

For agreement with Dr. Peirce's observations, Ebstein's theory of gout, "that it is a species of coagulation necrosis in which urates are deposited in the necrotic area," is suggested.

Garrod's experiment for the presence of uric acid in the blood serum should be applicable if not diagnostic.

As Dr. Kirk has said, the analyses quoted by Dr. Allan contain an element of fallacy; urates, as urates, would through such means be decomposed and give reactions not as urates, but as carbonates. One analysis simply resolves the deposits into individual chemical elements, from which no conclusion can be deduced.

Dr. Peirce and Dr. Truman seem to give different definitions as to the local disease we are discussing, Dr. Truman's bearing the same relation to Dr. Peirce's that the renal effect of a large dose of turpentine bears to chronic nephritis. If the gouty diathesis view be true as a cause of disease in the peridental membrane, we should expect to find the disorder prevalent among brewery-men, who consume large quantities of malt liquors. Gout is a common disease among English porter drinkers. Is phagedenic pericementitis also? that is a question. Dr. Allan's classification into pathogenic and traumatic luxation is too indefinite; it includes but does not place this particular affection.

The form in which we are likely to see this disorder would be in the condition known as lithæmia.

Certainly decided evidence has been presented this evening to show the connection between attacks of pericementitis and the ingestion of undue amounts of nitrogenous food. This does not prove that carbohydrates form no disturbing element; they may act in the production of the gastric catarrh. The exclusive use of certain foods will produce another degenerative disorder, not often seen, —that is, scorbutus. Not that there is any similarity, but it is an evidence of the effect of diet upon health.

Adjourned.

